# Towards universal deconvolution of tissue‐level multiomics for translational medicine

**DOI:** 10.1002/ctm2.70826

**Published:** 2026-09-11

**Authors:** Renjie Liu, Tianyi Zhao, Yadong Wang

**Affiliations:** ^1^ Faculty of Computing Harbin Institute of Technology Harbin China; ^2^ Zhengzhou Advanced Research Institute of Harbin Institute of Technology Zhengzhou China; ^3^ School of Medicine and Health Harbin Institute of Technology Harbin China

1

Over the past several decades, large‐scale population cohorts profiled using transcriptomics, proteomics, and metabolomics have continued to accumulate, enabling the discovery of disease‐associated molecular features, biomarkers, and therapeutic targets. These cohorts remain indispensable resources for studying human disease.^1^ However, tissue‐level omics data represent averaged molecular signals generated by multiple‐cell populations and therefore conflate changes in cellular composition with alterations in the intrinsic states of individual cell types.^2^ Single‐cell sequencing technologies have enabled disease heterogeneity to be investigated at the levels of both cell type and cell state. Nevertheless, their application to large‐scale longitudinal clinical cohorts remains constrained by cost, tissue preservation conditions, sample‐processing requirements, and batch effects. Moreover, many archived clinical specimens cannot be reprocessed into viable single‐cell suspensions. The central challenge is therefore not to replace existing tissue‐level cohorts with single‐cell sequencing, but to use limited single‐cell references to translate existing and newly generated tissue‐level omics data into interpretable cellular phenotypes.

Deconvolution provides an important bridge between these two levels of measurement. By using single‐cell data as a reference, deconvolution methods estimate the relative abundance of different cell types or cell states from tissue‐level molecular profiles. Transcriptomic deconvolution methods, exemplified by CIBERSORTx,^3^ have been widely applied to large clinical cohorts.^4^ Modern clinical studies, however, increasingly generate transcriptomic, proteomic, and metabolomic data, whereas most existing deconvolution methods have been designed for a specific omics modality. Differences in their distributional assumptions, feature spaces, and error structures make cellular composition estimates obtained from different modalities and cohorts difficult to compare directly.

DECODE^5^ combines adversarial domain alignment with noise‐aware representation learning to provide a unified framework for the deconvolution of transcriptomic, proteomic, and metabolomic data (Figure 1a). This represents a conceptual shift in the field: researchers no longer need to select separate deconvolution methods for cohorts profiled using different omics modalities, while the framework can, to some extent, accommodate differences in biological states between the single‐cell reference and the target cohort. By projecting cohorts from different omics modalities into a shared cell‐abundance space, DECODE enables their joint analysis, effectively increasing the analytical cohort size and statistical power while improving the representation of rare phenotypes across studies (Figure 1b). This allows associations between phenotypes and cell‐type proportions to be interpreted with greater confidence. These capabilities highlight the clinical value of DECODE in transforming existing molecular cohorts into interpretable cellular phenotypes for biomarker discovery, patient stratification, and investigation of the mechanisms underlying therapeutic response (Figure 1c).

**FIGURE 1:**
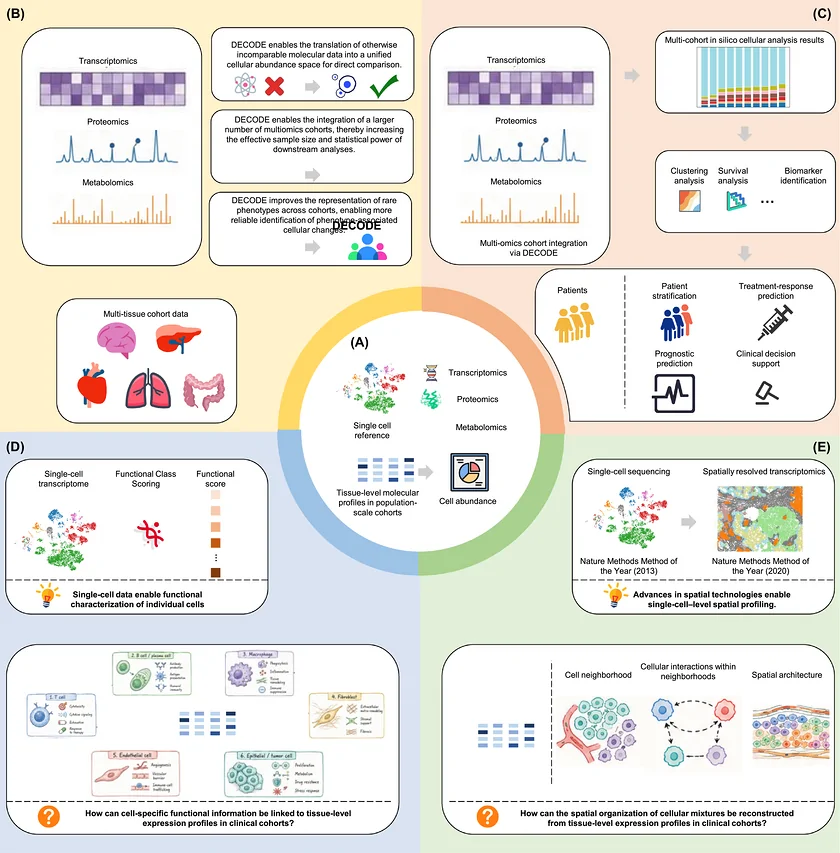
From multi‐omics deconvolution to functional and spatial cellular phenotyping in large clinical cohorts. (a) Overview of DECODE, which leverages single‐cell references to infer cellular composition from tissue‐level molecular profiles. (b) By mapping transcriptomic, proteomic and metabolomic measurements into a unified cellular‐abundance space, DECODE facilitates cross‐omics and cross‐cohort comparison, increases the effective sample size available for downstream analyses and improves the representation of rare phenotypes across heterogeneous cohorts and tissues. (c) Integration of cellular profiles inferred from multiple molecular cohorts enables in silico cellular analyses, including patient clustering, survival analysis and biomarker identification, with potential applications in patient stratification, treatment‐response prediction, prognosis and clinical decision support. (d) A future direction is to extend deconvolution from cell‐type abundance to cell‐specific functional phenotypes. Functional scoring of single‐cell data can quantify biological programmes and response states within individual cells; linking such information to tissue‐level molecular profiles could enable functional cellular phenotyping across large clinical cohorts. (e) A complementary direction is to recover spatial organisation from tissue‐level molecular data. Advances in spatial omics enable characterisation of cellular neighbourhoods, cell–cell interactions and higher‐order tissue architecture, motivating the development of approaches that transfer spatial information from spatial references to large cohorts lacking direct spatial measurements.

Cellular composition represents only one dimension of the disease ecosystem. Two patients may have similar proportions of immune cells while those cells exhibit markedly different activation, exhaustion, inflammatory, or immunosuppressive programmes.^6^ Although DECODE can estimate the abundance of predefined cell states, the proportion of a cell‐state subgroup is not equivalent to patient‐specific functional activity within a particular cell type. Abundance deconvolution alone therefore cannot fully answer the clinically important question of ‘which pathway is altered in which cell type in a given patient’. The next generation of deconvolution methods should extend beyond abundance estimation to reconstruct cell‐type‐specific gene expression, protein activity, metabolic programmes, signalling pathways, and drug‐response states (Figure 1d).^7^ Such function‐resolved deconvolution must explicitly distinguish changes in cell abundance from alterations in intrinsic cellular function and should be evaluated using paired tissue‐level and single‐cell measurements or other orthogonal experimental evidence.

Spatial omics introduces a third dimension to this analytical framework. Disease progression not only alters cellular composition and function but also reorganises tumour margins, immune aggregates, fibrotic regions, and other tissue niches.^8^ Spatial omics technologies can characterise these structures directly, but their high cost currently limits their application to large clinical cohorts. Because conventional tissue‐level data do not contain spatial coordinates, deconvolution cannot recover the precise locations of individual cells or reconstruct the complete topology of a tissue. A more feasible objective is to use spatial omics data to define biologically meaningful spatial compartments or niches and subsequently infer the relative abundance or functional activity of these reference‐defined structures from tissue‐level cohort data (Figure 1e). This strategy could enable spatial features discovered in small‐scale studies to be tested across much larger clinical cohorts. However, the inferred spatial phenotypes would still require validation using multiregional sampling, histopathological images, or independent spatial omics data.

The ongoing evolution of deconvolution should therefore be viewed as more than a continuous improvement in the accuracy of cell‐proportion estimation. It represents a fundamental expansion of the target output: from determining which cells are present in a tissue to understanding what those cells are doing and how they are organised into disease‐associated niches. DECODE establishes a foundation for cross‐omics analysis of cellular composition and predefined cell states. Future integration of cell‐type‐specific functional programmes and reference‐defined spatial structures into this framework could transform large‐scale tissue‐level omics cohorts into multidimensional cellular phenotype resources. Such resources may provide more mechanistically informative evidence for disease subtyping, patient stratification, therapeutic‐response prediction, investigation of drug‐resistance mechanisms, and prioritisation of candidate therapeutic targets.

## AUTHOR CONTRIBUTIONS

Renjie Liu and Tianyi Zhao jointly conceived the manuscript and contributed to its writing. Yadong Wang supervised the overall process.

## FUNDING

Tianyi Zhao was supported by the National Natural Science Foundation of China (grant no. 62572148).

## CONFLICT OF INTEREST STATEMENT

The authors declare no competing interests.

## ETHICS STATEMENT

This article does not involve any new studies with human participants or animals conducted by the authors.

## References

[1] Yurkovich JT , Evans SJ , Rappaport N , et al. The transition from genomics to phenomics in personalized population health. Nat Rev Genet. 2024;25:286‐302. doi:10.1038/s41576‐023‐00674‐x 38093095 10.1038/s41576-023-00674-x

[2] Garmire LX , Li Y , Huang Q , et al. Challenges and perspectives in computational deconvolution of genomics data. Nat Methods. 2024;21:391‐400. doi:10.1038/s41592‐023‐02166‐6 38374264 10.1038/s41592-023-02166-6

[3] Newman AM , Steen CB , Liu CL , et al. Determining cell type abundance and expression from bulk tissues with digital cytometry. Nat Biotechnol. 2019;37:773‐782. doi:10.1038/s41587‐019‐0114‐2 31061481 10.1038/s41587-019-0114-2 PMC6610714

[4] Zeng AGX , Bansal S , Jin L , et al. A cellular hierarchy framework for understanding heterogeneity and predicting drug response in acute myeloid leukemia. Nat Med. 2022;28:1212‐1223. doi:10.1038/s41591‐022‐01819‐x 35618837 10.1038/s41591-022-01819-x

[5] Zhao T , Liu R , Sun Y , et al. DECODE: deep learning‐based common deconvolution framework for various omics data. Nat Methods. 2026;23:596‐608. doi:10.1038/s41592‐026‐03007‐y 41772096 10.1038/s41592-026-03007-y PMC12982128

[6] Lopez De Rodas M , Villalba‐Esparza M , Sanmamed MF , Chen L , Rimm DL , Schalper KA . Biological and clinical significance of tumour‐infiltrating lymphocytes in the era of immunotherapy: a multidimensional approach. Nat Rev Clin Oncol. 2025;22:163‐181. doi:10.1038/s41571‐024‐00984‐x 39820025 10.1038/s41571-024-00984-x

[7] Fang Z , Liu X , Peltz G . GSEApy: a comprehensive package for performing gene set enrichment analysis in Python. Bioinformatics. 2023;39(1): btac757. 36426870 10.1093/bioinformatics/btac757 PMC9805564

[8] Mo C‐K , Liu J , Chen S , et al. Tumour evolution and microenvironment interactions in 2D and 3D space. Nature. 2024;634:1178‐1186. doi:10.1038/s41586‐024‐08087‐4 39478210 10.1038/s41586-024-08087-4 PMC11525187

